# Malrotation of Kidney Ectopia and Spontaneous Renal Vein Thrombosis

**DOI:** 10.7759/cureus.99785

**Published:** 2025-12-21

**Authors:** Amani Alabdouli, Sonia Lamichhane, Thiagarajan Jaiganesh

**Affiliations:** 1 Emergency Medicine, Tawam Hospital, Al Ain, ARE

**Keywords:** abdominal pain, acute abdomen, kidney malrotation, renal ectopia, renal vein thrombosis, spontaneous thrombosis

## Abstract

Renal ectopia and malrotation are uncommon congenital anomalies that arise from abnormal kidney ascent and rotation during embryologic development. Although often discovered incidentally, these structural variations may alter venous drainage and predispose to stasis and thrombosis. Renal vein thrombosis (RVT) is rare in adults and usually occurs secondary to nephrotic syndrome, malignancy, dehydration, or hypercoagulable disorders. The coexistence of renal malrotation, ectopia, and spontaneous RVT in an otherwise healthy adult is exceptionally unusual.

A 35-year-old woman presented with severe right-lower-quadrant abdominal pain, nausea, and fever, initially raising concern for appendicitis. Laboratory results revealed severe microcytic anemia with reactive thrombocytosis. Contrast-enhanced computed tomography demonstrated a malrotated ectopic left kidney located in the iliac fossa with associated left RVT. Comprehensive evaluation for thrombophilia, autoimmune disease, and malignancy was negative, while iron studies confirmed marked iron-deficiency anemia. The patient was treated with blood transfusion and anticoagulation, resulting in complete clinical and radiologic resolution after one year of follow-up.

This case illustrates how congenital renal vascular anomalies and hematologic abnormalities may interact to produce thrombosis. Altered venous outflow from the ectopic kidney likely caused local stasis, while iron-deficiency anemia contributed to a transient hypercoagulable state through reactive thrombocytosis and endothelial dysfunction. Clinicians should consider RVT in the differential diagnosis of atypical abdominal or flank pain when imaging reveals renal positional anomalies. Early recognition and anticoagulation can preserve renal function and prevent unnecessary surgical intervention.

## Introduction

The kidneys develop in the pelvis and ascend to the lumbar region between the sixth and ninth weeks of gestation, rotating medially so that the hilum faces anteromedially. Arrest or abnormal rotation during this process results in renal ectopia or malrotation. The incidence of renal ectopia is approximately one in 1000 live births, while malrotation occurs in roughly one in 2000 individuals [1]. Most cases are asymptomatic and discovered incidentally, but altered position and aberrant vasculature can predispose to urinary obstruction, infection, or vascular stasis.

Renal vein thrombosis (RVT) is rare in adults and typically occurs secondary to nephrotic syndrome, dehydration, malignancy, or thrombophilia [2]. Altered venous drainage in an ectopic or malrotated kidney may promote local venous stasis and increase the risk of thrombosis. RVT can mimic appendicitis or renal colic, and prompt imaging is essential for diagnosis [2,3]. This report presents a rare coexistence of renal ectopia with malrotation and spontaneous RVT in a healthy young adult, illustrating the importance of imaging and recognition of subtle predisposing factors such as severe iron-deficiency anemia (IDA).

## Case presentation

A 35-year-old previously healthy woman presented with severe right lower-quadrant (RLQ) abdominal pain for three days, associated with nausea and low-grade fever. On examination, she had RLQ tenderness and guarding; vital signs were as follows: temperature 37°C, blood pressure 110/72 mmHg, pulse 99 bpm, respiratory rate 18/min.

Analgesia with intravenous paracetamol and ketorolac was administered, followed by tramadol and antiemetics for persistent pain. Pelvic ultrasound excluded ovarian torsion. Because the clinical picture suggested appendicitis, a contrast-enhanced CT scan was performed.

Laboratory results (Table 1) revealed leukocytosis (11.7 × 10⁹/L) and severe anemia (hemoglobin 4.7 g/dL). β-hCG and urinalysis were normal. The patient denied hematuria, dysuria, or gastrointestinal bleeding.

**Table 1:** Key laboratory findings demonstrating iron-deficiency anemia and normal coagulation profile.

Parameter	Result	Reference Range	Interpretation
Hemoglobin	4.7 g/dL	12-16 g/dL	Severe anemia
WBC	11.7 × 10⁹/L	4-10 × 10⁹/L	Mild leukocytosis
Platelets	540 × 10⁹/L	150-400 × 10⁹/L	Reactive thrombocytosis
Serum iron	1.3 µmol/L	10-30	↓
Ferritin	9 ng/mL	13-150	↓
Transferrin	3.55 g/L	2-3.6	↑
Transferrin sat.	2 %	20-50 %	↓
PT/aPTT/INR	12.8 s/32 s/1.0	-	Normal
Protein C/S/AT III	Normal	-	Normal
Factor V Leiden, LA, ACL, β2GPI	Negative	-	Normal

**Figure 1:**
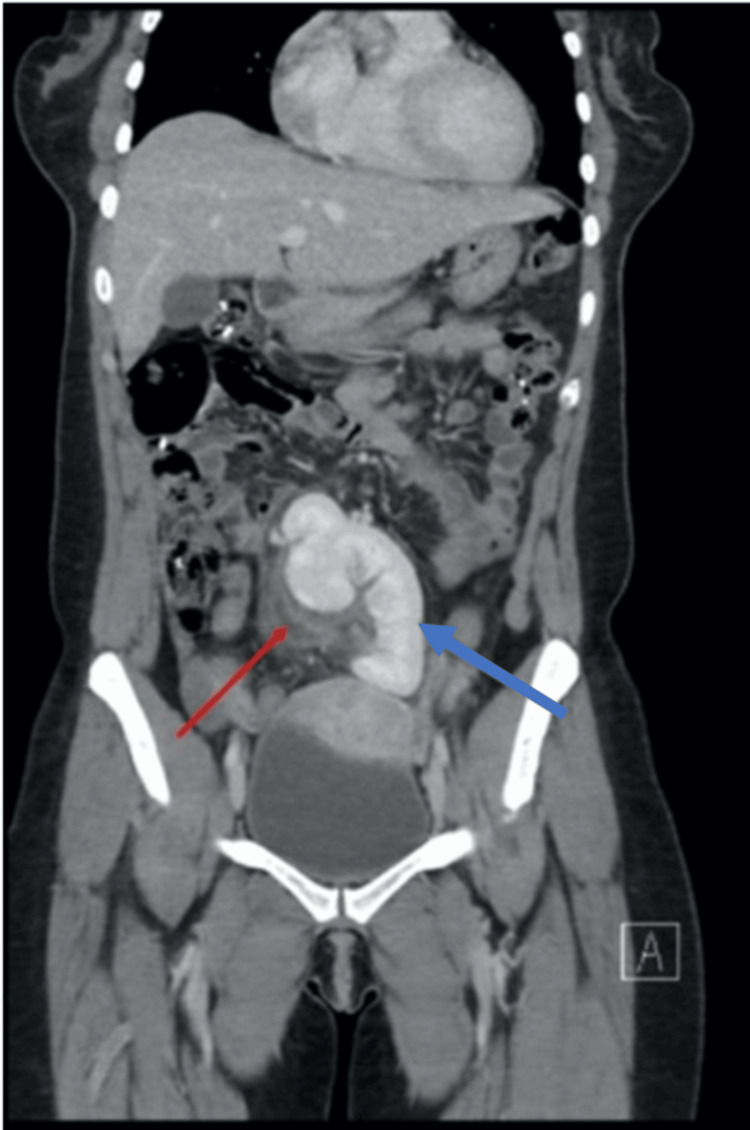
Coronal contrast-enhanced computed tomography (CT) of the abdomen demonstrates malrotation and ectopia of the left kidney, which is visualized in the midline of the lower abdomen (blue arrow). A filling defect within the left renal vein (red arrow) is seen, consistent with renal vein thrombosis.

A contrast-enhanced computed tomography (CT) scan of the abdomen and pelvis revealed malrotation and ectopia of the left kidney, which was located in the midline of the lower abdomen rather than its normal left retroperitoneal position. The CT demonstrated a filling defect within the left renal vein, consistent with RVT (Figure 1). No appendiceal or gynecologic pathology was identified. The patient was admitted, transfused with two units of packed red blood cells, and commenced on therapeutic anticoagulation.

Further investigations showed the following: Iron studies: serum iron 1.3 µmol/L ↓ (10-30), ferritin 9 ng/mL ↓ (13-150), transferrin 3.55 g/L ↑ (2-3.6), transferrin saturation 2 % ↓ (20-50) - confirming IDA.·Vitamin B12: 310 pg/mL; haptoglobin: 1.6 g/L (normal). Coagulation profile: PT 12.8 s, aPTT 32 s, INR 1.0; thrombophilia screen (protein C, protein S, antithrombin III, factor V Leiden, lupus anticoagulant, anticardiolipin, β-2 glycoprotein antibodies, homocysteine) - all normal. Albumin, tumor markers, autoimmune, and viral screens were negative. PET-CT and upper-GI endoscopy were unremarkable.

She was discharged on oral anticoagulation with hematology follow-up. Six-month imaging confirmed recanalization of the renal vein, and therapy for 12 months.

## Discussion

Renal malrotation and ectopia are rare congenital anomalies that result from disturbances in kidney ascent and rotation during embryogenesis. Normally, between the sixth and ninth weeks of gestation, the metanephric kidneys migrate upward from the pelvis to their final retroperitoneal position while rotating medially. Disruptions in vascular remodeling or mechanical constraints can hinder this process, producing kidneys that are both ectopic and malrotated [1]. These anomalies are often discovered incidentally but may predispose to complications such as urinary stasis, infection, calculi, or vascular compromise [1,2].

In the present case, the left kidney was both malrotated and ectopic, located in the midline of the lower abdomen. This abnormal position likely resulted in altered venous drainage and local hemodynamic changes. The left renal vein, which normally drains horizontally into the inferior vena cava, may have been partially compressed or experienced flow turbulence due to its aberrant orientation. Such venous stasis creates favorable conditions for thrombus formation even in the absence of systemic thrombophilia [2,3]. Therefore, the patient’s congenital renal vascular configuration is a plausible predisposing factor for spontaneous RVT.

RVT in adults is uncommon and typically secondary to nephrotic syndrome, malignancy, or systemic hypercoagulable disorders [2,3]. When occurring without such factors, as in this case, diagnosis can be difficult because symptoms such as abdominal or flank pain, nausea, and fever are nonspecific and can mimic appendicitis, pyelonephritis, or gynecologic conditions. Imaging, therefore, plays a pivotal role. The contrast-enhanced CT in this case revealed the key finding: a filling defect within the left renal vein associated with the ectopic kidney, confirming the diagnosis and preventing unnecessary surgical exploration.

A notable feature of this case is the coexistence of severe IDA, which may have contributed to thrombus formation. Although anemia is generally associated with decreased viscosity, iron deficiency paradoxically promotes thrombosis through reactive thrombocytosis, endothelial dysfunction, and increased fibrinogen levels [4-6]. The patient’s markedly low hemoglobin, elevated platelet count, and absence of inherited or acquired thrombophilia support this mechanism. Emerging studies have shown that correction of iron deficiency reduces thrombosis risk, underscoring the importance of recognizing IDA as a transient hypercoagulable state [4-6].

Management of RVT depends on etiology and extent. Prompt anticoagulation remains the cornerstone of therapy, with the goal of preventing thrombus propagation and preserving renal function [2,3]. Our patient responded well to anticoagulation and transfusion, and renal function remained normal. Unfortunately, she left the UAE before completing long-term follow-up, limiting documentation of radiologic resolution. Nonetheless, early diagnosis and treatment likely prevented complications such as renal impairment or thrombus extension.

This case highlights the complex interplay between congenital renal vascular anomalies and hematologic abnormalities in the pathogenesis of RVT. Clinicians should maintain a high index of suspicion for vascular etiologies of abdominal or flank pain, particularly when imaging reveals positional renal anomalies. A multidisciplinary approach involving radiology, hematology, and nephrology is essential for accurate diagnosis and optimal management.

## Conclusions

This case underscores the rare coexistence of renal ectopia with malrotation and spontaneous RVT in an otherwise healthy adult. Altered venous anatomy likely promoted local stasis, while severe IDA with reactive thrombocytosis contributed to transient hypercoagulability. Normal coagulation results excluded systemic thrombophilia. Clinicians should maintain a high index of suspicion for vascular causes of abdominal pain when imaging reveals renal anomalies. Early recognition and anticoagulation are crucial for renal preservation and excellent outcomes.
